# Observation of curative effect on meridian theory-based extracorporeal shock wave therapy for non-specific low back pain: study protocol for a randomized controlled trial

**DOI:** 10.1186/s13018-022-03146-w

**Published:** 2022-05-13

**Authors:** Yongfu Fan, Feilai Liu, Mengna Li, Xiaodi Ruan, Mingli Wu, Kaiqi Su, Jing Gao, Xiaodong Feng

**Affiliations:** 1 Henan University of Chinese Medicine, Zhengzhou, 450000 China; 2 Rehabilitation Center, The First Affiliated Hospital of Henan University of Chinese Medicine, 19# Renmin Road, Jinshui District, Zhengzhou, 450000 Henan Province China; 3 Institute of Pain Medicine and Special Environmental Medicine, Nantong University, Nantong, 226019 China

**Keywords:** Extracorporeal shock wave therapy (ESWT), Meridian theory, Non-specific low back pain (NLBP), Clinical trial

## Abstract

**Background:**

Non-specific low back pain (NLBP) is a major global socioeconomic burden, and the prevalence of NLBP is still on the rise. At present, there is no ideal drug to cure this disease. This may be the reason why patients often use complementary therapies. Among them, extracorporeal shock wave therapy (ESWT) has gradually received more attention and has become the main treatment method for NLBP. The purpose of this study is to provide scientific evidence for the effect and safety of meridian theory-based ESWT on NLBP.

**Objective:**

This study aims to evaluate the effect and safety of meridian theory-based ESWT on NLBP. This study will also provide more high-quality experimental evidence for the clinical application of meridian theory-based ESWT for the treatment of NLBP in future.

**Methods:**

The study design is a single-blind, multi-center, randomized controlled trial. 66 patients with NLBP, aged 18 to 60 years, will be randomly divided into two groups: the experimental group (*N* = 33), which will receive meridian theory-based ESWT application, and the control group (*N* = 33) which will receive conventional ESWT treatment. These two applications will be carried out twice a week for two weeks. The primary outcome will be the Visual Analog Scale (VAS), and the secondary outcomes will be Oswestry Disability Index (ODI), Surface Electromyography (sEMG), and Patient Health Questionnaire-15 (PHQ-15). All outcomes will be evaluated at baseline and after the intervention (7 days, 14 days).

**Discussion:**

Results of this trial will contribute to providing rigorous clinical evidence for the efficacy and security of meridian theory-based ESWT for NLBP.

*Trial registration*: Chinese Clinical Trial Registry, ChiCTR2100051049. Registered on 10 September 2021, http://www.chictr.org.cn/showproj.aspx?proj=46316.

## Background

Low back pain is the major cause of dysfunction, affecting all age groups and being more common in adults [1, 2]. At the same time, the prevalence and burden of low back pain increase with age [3]. Many patients with low back pain cannot be specifically diagnosed, and approximately 85% of cases cannot be attributed to specific pathological changes or nerve root irritation [4]; therefore, most patients with low back pain have NLBP [5]. Pain and dysfunction are common features of NLBP [6, 7].

At present, most treatments for NLBP have limited efficacy and a high recurrence rate [8]. The main purpose of clinical treatment of NLBP is to relieve pain and improve the quality of life of patients. There are many clinical treatments for NLBP, including conservative therapy and surgery [9–11]. Conservative therapy including bed rest, drug therapy, physical exercise, and exercise therapy can relieve NLBP to a certain extent, but there are also many drawbacks; Surgical treatment is accompanied by problems such as postoperative infection and poor long-term efficacy, and the high cost brings a heavy economic burden to patients [12]. Therefore, there is an urgent need for a safe, effective, and economical treatment for NLBP.

ESWT has been extensively used for the treatment of NLBP in recent years [13, 14]. ESWT is a therapeutic method that applies shock waves to lesions from the inner body to boost blood flow and stimulate or reactivate affected connective tissue (including tendons and bones) to relieve pain and improve dysfunction [15]. Recently, based on the meridian theory of Traditional Chinese Medicine(TCM), ESWT combined with acupoint stimulation has played a therapeutic role [16]. The purpose of this study is to observe the clinical efficacy of meridian theory-based ESWT on pain and dysfunction in NLBP patients.

## Methods

### Study design

**Fig. 1:**
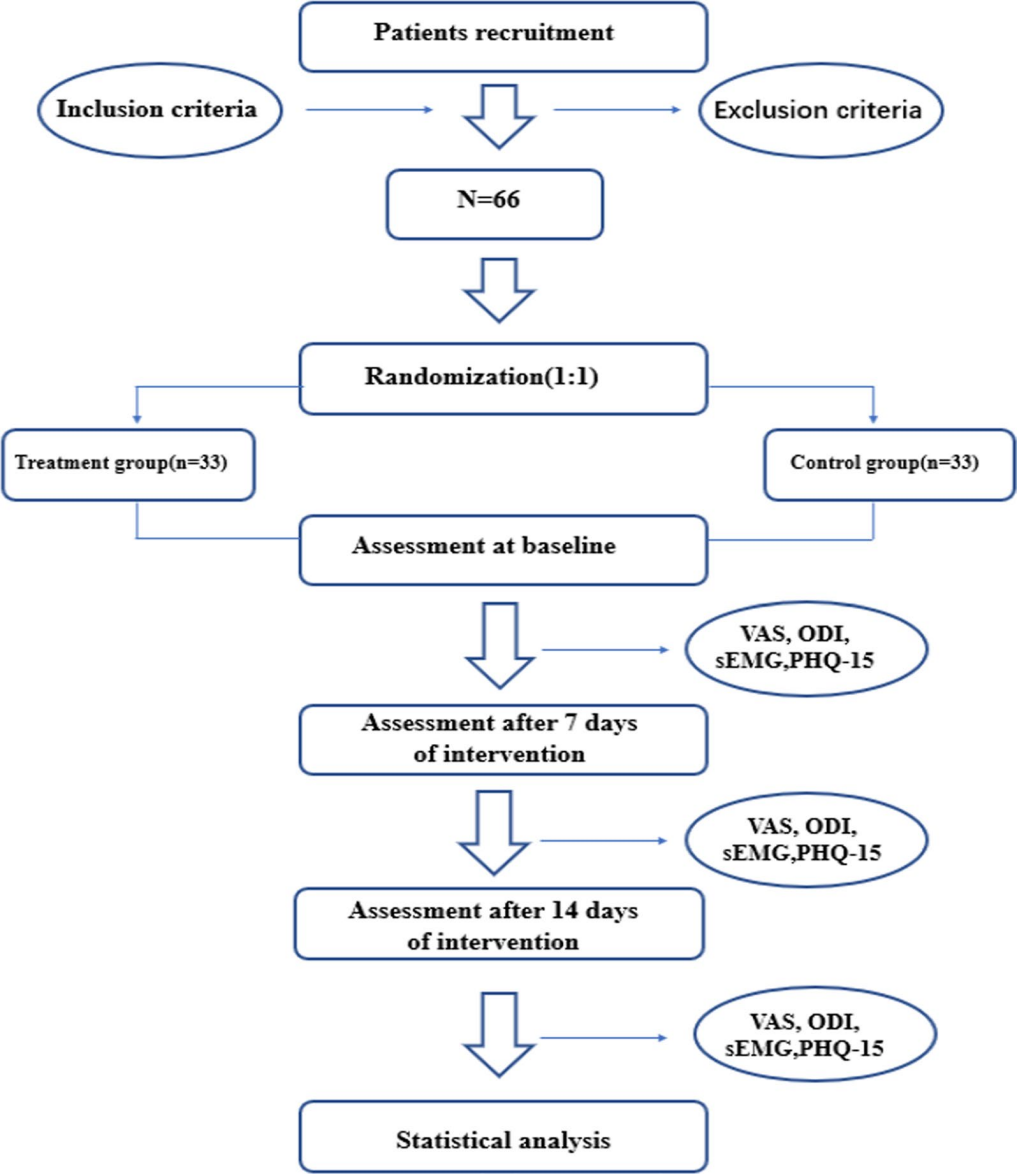
Flowchart of the trial

This is a single-blind, multi-center, randomized placebo-controlled trial supported by Henan Provincial Administration of TCM. The trial will be jointly conducted by three centers in Zhengzhou, China: the First Affiliated Hospital of Henan University of CM, Henan Provincial Hospital of CM, and the Third Affiliated Hospital of Henan University of CM. Patients between 18 and 60 years of age with NLBP will be included. When participants are eligible for inclusion after being informed about the purpose and procedures of the study, they will be asked to sign an informed consent form. Then, Sixty-six patients will be recruited and randomly assigned to two groups: the experimental group (*N* = 33) and the control group (*N* = 33). Participants in the experimental group will receive ESWT based on meridian theory and participants of the control group will receive conventional ESWT treatment. Patients will be treated twice a week for two weeks. All the participants will be assessed 3 times: baseline (evaluation before treatment), the middle of the treatment (7 days after treatment starts), and the end of the treatment (14 days after treatment starts). The volunteers will complete the VAS, ODI, sEMG, and PHQ-15 assessments. The study flowchart is shown in Fig. 1. The trial process chart is shown in Table 1.

**Table 1:** Timing of treatment assessments and data collection

Timepoint	Study period			
	Enrolment	Baseline	Treatment phase	
	− 1 week	0 week	1 week	2 weeks
Enrolment				
Eligibility screen	×			
Informed consent	×			
Medical history	×			
Merger disease	×			
Randomization		×		
Interventions				
Experimental group		×	×	×
Control group		×	×	×
Assessments				
VAS	×	×	×	×
ODI		×	×	×
sEMG		×	×	×
Safety evaluation		×	×	×
Adverse events		×	×	×

### Ethics committee approval

This trial was reviewed by the ethics committee of the First Affiliated Hospital of Henan University of CM on August 24, 2021 (reference number: 2021HL-203-01). Written informed consent will be obtained from all participants. The study was registered with the Chinese Clinical Trial Registry (Identifier ChiCTR2100051049.)

### Participants

The main participants will be recruited independently through the outpatient and inpatient systems of three hospitals in China—(the First Affiliated Hospital of Henan University of CM, Henan Provincial Hospital of CM, and the Third Affiliated Hospital of Henan University of CM). To recruit participants, three independent centers will publish individually online study advertisements through hospital websites and WeChat and we will also put up posters and distribute leaflets offline in crowded areas of the hospital. The recruitment of participants began on 15 September 2021 and is expected to end on 9 December 2022, or it may be completed earlier if a sufficient number of patients are recruited.

### Inclusion criteria

Participants meeting the following criteria will be included:

1. Male or female patients aged 18–60;
2. Low back pain that persists for more than three months without a specific cause;
3. VAS score greater than 3 points;
4. Patients with normal intelligence and language skills;
5. NLBP patients who have not received any treatment in the past two weeks;
6. Agree to receive only ESWT-related treatments during treatment;
7. Willing to volunteer for the trial and provide informed consent.

### Exclusion criteria

Participants who report any of the following will be excluded:

1. Patients will be excluded when low back pain is caused by specific pathological factors such as tumors, Osteoporosis, lumbar fractures, rheumatoid arthritis;
2. The lower extremities are affected by neurological malfunction due to NLBP;
3. Known or suspected serious congenital or acquired spinal pathology, lumbar disc herniation with nerve symptoms, spinal deformity, spondyloarthropathy, etc.;
4. Patients with other serious diseases such as immune diseases, endocrine diseases, and abnormal liver and kidney functions;
5. Patients deemed by researchers as ineligible to participate due to severely abnormal/unstable laboratory test results or vital signs;
6. ESWT -phobic patients.

### Elimination criteria

1. Patients who do not meet the diagnostic criteria for NLBP and are erroneously included;
2. Patients who are judged by the researchers as unsuitable to continue the trial because of serious adverse complications.

### Randomization

Participants who are eligible in the screening phase and agree to sign the consent form will be randomly assigned to the experimental group or the control group. The random sequence numbers will be generated through the computer. The allocation will be concealed using consecutively numbered sealed and opaque envelopes. For this, an independent researcher who will not participate in other study procedures will carry out the randomisation process to avoid bias. The allocation sequence will be generated by a specialized statistician who does not participate in this experiment. Relevant independent researchers in three centres will recruit participants and assign them to two different intervention groups.

### Blinding

This is a single-blind study. All other researchers, such as assessors and data analysts, will be unaware of group assignments. To confirm whether participants achieved blinding, assessors will use a "blinding test" questionnaire to ask participants what type of treatment they think they received after the first and final treatment.

### Sample size calculation

We will conduct this study in a randomized controlled trial design method and the main observed outcome was the improvement of patients’ pain. Previously, we conducted a small sample(12 cases) of clinical observation and found that the 1-week experimental group received ESWT with meridian theory-based and the control group received conventional ESWT treatment can reduce the level of the VAS scale by 3.45 ± 1.31 and 2.33 ± 1.13, respectively. The significance test level was 0.05, and the test power was 0.9. The sample size was calculated by using:

$$N = 2 \times \left[ {\left( {z_{\alpha } + z_{\beta } } \right) \times \sigma /\delta } \right]^{2}$$

*N* is the required sample size for each group, and the sample size of each group is equal. Where *α* is 0.05 and *β* is 0.1, the normal distribution quantile table shows that:

$$Z_{{\alpha /{2}}} = {1}.{96},\;{\text{and}}\;Z_{\beta } = {1}.{282}.$$

*σ* and *δ* represent the population standard deviation and allowable error, which are 1.31 and 1.12, respectively. By substituting the above data into the formula, 29 cases will be needed in each group. Taking into account the 10% expulsion rate, a total sample of 66 participants will be sufficient.

### Interventions

The intervention treatment period will include 4 sessions during 2 weeks. Licensed medical doctors who are bothering at least 4 years of clinical experience will perform the intervention, and we will conduct standardized training for the clinicians who implement the treatment in the three research centers before the treatment and try to maintain the consistency of the research. Meanwhile, each patient will receive the same routine therapeutic drug and rehabilitation. The interventions for the two groups are as follows:

**Routine therapeutic drug treatment:** All patients will use blood pressure control, blood lipid adjustment, blood sugar control, and nutritional nerve drug as routine therapeutic drug treatment following the physicians' instructions.

**Extracorporeal shock wave intervention:** ESWT will be applied by a BTL-5000 pneumatic ballistic ESWT machine, 8.0–10 Hz, 1.6–3.0 bar energy density, 2000 pulses, twice a week for two weeks.

**The experimental group:** In addition to the routine treatment for patients with low back pain, ESWT will also be performed at Huantiao(GB30), Baihuanshu(BL30), Xiaochangshu(BL27), Pangguangshu(BL28), Guanyuanshu(BL26), Qihaishu(BL24), Dachangshu(BL25), Sanjiaoshu(BL22), Chengfu(BL36), Yaoyan(EX-B7) and Yaoyi (EX-B6) in the experimental group [17] (Table 2).

**Table 2:** Locations of acupoints for ESWT

Acupoints	Location
Huantiao (GB30)	In the buttocks region, at the junction of the lateral one-third and medial two-thirds of the line connecting the prominence of the greater trochanter with the sacral hiatus
Baihuanshu (BL30)	In the sacral region, at the same level as the fourth posterior sacral foramen, 1.5 B-cun lateral to the median sacral crest
Xiaochangshu (BL27)	In the sacral region, at the same level as the first posterior sacral foramen, and 1.5 B-cun lateral to the median sacral crest
Pangguangshu (BL28)	In the sacral region, at the same level as the second posterior sacral foramen, and 1.5 B-cun lateral to the median sacral crest
Guanyuanshu (BL26)	In the lumbar region, at the same level as the inferior border of the spinous process of the fifth lumbar vertebra (L5), 1.5 B-cun lateral to the posterior median line
Qihaishu (BL24)	In the lumbar region, at the same level as the inferior border of the spinous process of the third lumbar vertebra (L3), 1.5 B-cun lateral to the posterior median line
Dachangshu (BL25)	In the lumbar region, at the same level as the inferior border of the spinous process of the fourth lumbar vertebra (L4), 1.5 B-cun lateral to the posterior median line
Sanjiaoshu (BL22)	In the lumbar region, at the same level as the inferior border of the spinous process of the first lumbar vertebra (L1), 1.5 B-cun lateral to the posterior median line
Chengfu (BL36)	In the buttock region, at the midpoint of the gluteal fold
Yaoyan (EX-B7)	In the lumbar region, au niveau du rebord inferieur du processus epineux L4, lateral a la ligne mediane posterieurede 3, 5 cun
Yaoyi (EX-B6)	In the lumbar region, au niveau du rebord inferieur du processus epineux L4, lateral a la ligne mediane posterieurede 3 cun

**The control group:** The patient will be placed in the prone position to expose below the seventh rib and above the gluteal groove. The clinician will use the thumbs of both hands to press and palpate from the middle to the sides with the strength that the patient can bear, and then the doctor will mark the pressure pain points with a marker according to the shape of the muscle. ESWT will then be performed at the tender point of the control group.

### Outcome measures

#### Primary outcome

**Visual Analog Scale (VAS):** Pain intensity will be assessed using VAS [18]. This questionnaire has good reliability and validity,and has the advantages of simplicity, time-saving, convenient operation, and wide application to a wide range of people. On the paper was drawn a horizontal line 10 cm long. One end of the horizontal line indicated no pain, the other end indicated severe pain; the middle part indicated different degrees of pain. On the horizontal line, we let the patient mark the degree of pain based on the feeling of self. Many clinical trials for NLBP use VAS as their main outcome.

#### Secondary outcome

**Oswestry Disability Index (ODI):** The ODI will be a simple and comprehensive patient-rated outcome used to assess Lumbar spine dysfunction [19]. The ODI is used to measure NLBP-related dysfunction and it includes 10 questions about daily activities, including pain intensity, personal care, lifting, walking, sitting, standing, sleeping, sexual life, social life and traveling. The ODI has six response categories. Each item scores from 0 (better) to 5 (worst). Each score will be transferred into a 0 to 100 scale, with lower scores indicating lesser Lumbar spine dysfunction.

**Surface electromyography (sEMG):** The sEMG [20] evaluation is an objective method to obtain the electromyographic signal during muscle activity, which is achieved by placing surface electrodes on the muscle layer. It can perform a quantitative and qualitative analysis of the function of muscles and can study multiple muscles in the body at the same time. It has the advantages of innovativeness, safety and reliability, convenient operation, and objective quantification.

**Patient Health Questionnaire-15 (PHQ-15):** An assessment of somatization and thus the severity of its symptoms in clinical practice and research can be done using the PHQ-15, a brief, self-administered questionnaire [21]. Among 15 somatic symptoms in the PHQ-1 5, each symptom is scored from 0 to 2 ("not bothered at all" to "bothered a lot"). Results included functional status as assessed by the 20-item Short-Form General Health Survey (SF-20), self-reported sick days, and clinic visits, as well as symptom-related difficulty. All six subscales of SF-20 decremented stepwise as PHQ-15 somatic symptoms severity increased.

### Data management

Case report forms(CRFs) will record all information about participants truthfully, completely, accurately and promptly.All relevant information of participants will be kept strictly confidential. After the trial, the quality control team will strictly review the submitted CRFs and ensure their accuracy and completeness of the CRFs.

### Statistical analysis

We will intend to apply SPSS V.20 software to the statistical analysis of all results. The CI will be established at 95%, and the significance level at 0.05. Missing data refers to the use of actual observations without imputation when the dropout rate does not exceed 10%. The normality test will be applied to all data first. Then, mean ± SD will be used to describe the data that fit the normal distribution; medians and interquartile ranges will be used to represent central and discrete trends in quantitative data that do not fit the normal distribution. The t-test and analysis of variance(ANOVA) will be applied to quantitative data, and the rank-sum test will be applied to count data to compare differences between groups. Sensitivity analysis will be carried out if necessary. *P* < 0.05 will be considered statistically significant.

### Withdrawal and dropout

The patients will be excluded from the trial if they withdraw their consent or lost follow-up, or if the patient is judged unable to continue participating. The researchers will document in detail why the trial was interrupted and whether each participant completed the trial.

### Quality control

Before the trial, all medical workers will accept strict training on related tasks. The therapist in this trial has licenses with at least 4 years of clinical experience. Monitors will inspect case report forms twice every week and patient situations during the treatment period. Dropouts and withdrawals involving the reasons will be documented detailedly during the trial. Participants’ information will be kept strictly secret at the study sites with limited access; Only investigators can view the data. All investigators will always maintain a strict privacy policy to protect confidentiality in the whole process of the trial.

### Safety evaluation and adverse events

The whole trial will monitor and report all adverse events (AEs). Any uncomfortable symptoms or disease during this study will be recognized as adverse events, such as localized hematoma, allergies, pain, dizziness, vomiting, or palpitations. The study will detail record AEs—including symptoms, onset, and end date, severity, relationship with ESWT, and outcome. Participants will stop treating if the treatments cause serious aggravation of symptoms and undergo related tests and treatments. Researchers will immediately report SAEs (e.g., requiring hospitalization, causing disability, or impaired ability to work) to the ethics committee of the First Affiliated Hospital of Henan University of CM and suspend the study.

## Discussion

The purpose of this study is to evaluate the efficacy of meridian theory-based ESWT in relieving pain and other symptoms which hurt the quality of life in these individuals with NLBP. To do this, the study involves the experimental group and the control group, and interventions will be conducted two times per week for 2 weeks. According to some previous studies [22], meridian theory-based ESWT can produce a faster and better analgesic effect than conventional ESWT. However, no studies have reported a difference in the effect of these two treatments in patients with NLBP. This trial comparing the two will fill gaps in the literature, thereby assisting physiotherapists and clinicians in clinical decision-making.

The VAS is one of the most commonly used measure tables for the assessment of pain and has been validated to detect changes in pain intensity [23]. The ODI scale is the most widely used dysfunction assessment questionnaire and has the advantages of simplicity, time savings, convenient operation, and relevance to a wide range of people [24]. The electromyographic signal measured by an sEMG is well suited for evaluating the effects of ESWT for NLBP [25]. Therefore, they have also been used in many studies applying ESWT for NLBP. Moreover, as both a screening tool and a measure of somatic symptom severity. PHQ-15 may be useful in clinical practice and research for checking for somatization and evaluating symptoms severity. These will support the main results and be meaningful for the overall efficacy assessment.

Strengths of the study include its strictly standardized endpoints, objective criteria, and strict quality control. The trial also has some limitations. First, this is a multicenter study conducted at three tertiary A hospitals in China and the results may not apply to primary hospitals or other countries. Second, the therapist will not be blinded due to the nature of the study, which might bring bias and influence the results. To achieve our clinical goals, we will strive to standardize every step of this study. We expect that the results of this trial will provide evidence of the efficacy and safety of meridian theory-based ESWT for NLBP. This will provide a practical and effective treatment method for the clinic, which has very important social significance.

## Data Availability

Not applicable; no data have yet been generated.

## Abbreviations

ESWT: Extracorporeal shock wave therapy
NLBP: Non-specific low back pain
VAS: Visual analog scale
ODI: Oswestry disability index
TCM: Traditional Chinese Medicine
sEMG: Surface electromyography
PHQ-15: Patient health questionnaire-15
CRFs: Case report forms
AEs: Adverse events

## Declarations

### Dissemination policy

After studying consummation, the final data will be submitted by the researcher to the Henan Administration of TCM in the form of a research report. Research results will be published in authoritative journals or presented at a high-level academic conference.
